# Advanced Spectroscopic and Theoretical Study and Assessment of Antimycotic Potential in a Synergistic Composition of a 1,3,4-Thiadiazole Derivative and Amphotericin B

**DOI:** 10.1021/acsomega.4c10113

**Published:** 2025-05-22

**Authors:** Lidia Ślusarczyk, Michaela Murzyniec, Mikołaj Gurba, Kamila Rachwał, Andrzej Górecki, James Hooper, Mariusz Gagoś, Arkadiusz Matwijczuk

**Affiliations:** † Department of Biophysics, Faculty of Environmental Biology, University of Life Sciences in Lublin, Akademicka 13, Lublin 20-950, Poland; ‡ Faculty of Chemistry, Jagiellonian University, Gronostajowa 2, Kraków 30-387, Poland; § Institute of Advanced Materials, Faculty of Chemistry, Wrocław University of Science and Technology, Wybrzeże Wyspiańskiego 27, Wrocław 50-370, Poland; ∥ Department of Biotechnology, Microbiology and Human Nutrition, Faculty of Food Science and Biotechnology, University of Life Sciences in Lublin, 8 Skromna Street, Lublin 20-704, Poland; ⊥ Department of Physical Biochemistry, Faculty of Biochemistry, Biophysics and Biotechnology, Jagiellonian University, Kraków 30-387, Poland; # Department of Cell Biology, Maria Curie-Sklodowska University, Akademicka 19, Lublin 20-033, Poland

## Abstract

The paper presents the results of an in-depth spectroscopic, theoretical (quantum chemical), and microbiological study conducted on a promising, synergistic composition of a newly considered 1,3,4-thiadiazole derivative, 1,3,4-thiadiazole: 2,4-dihydroxy-*N*-(5-methyl-1,3,4-thiadiazol-2-yl)­benzothioamide (TBTA), and the “gold standard” polyene antibiotic, amphotericin B (AmB). The spectroscopic properties of the system were extensively analyzed with a range of spectroscopic measurement techniques, including electronic fluorescence and absorption spectra, resonance light scattering measurements, circular dichroism spectra, dynamic light scattering, and fluorescence anisotropy, which were further complemented with time-resolved measurements of fluorescence lifetimes performed with the single-photon counting method. The samples were prepared in DMSO solutions and/or PBS buffer to facilitate observation of the monomeric, dimeric, and aggregated forms of the antibiotic previously identified in the literature. Absorption and fluorescence emission spectra measured for AmB and the synergistic composition revealed differences that indicated changes in AmB aggregation molecules, particularly in the buffer medium. Together with the results of the other spectroscopic techniques and computations, the effects of AmB disaggregation are clearly observed, and it is seen that TBTA interacts with AmB at the sites where other AmB molecules prefer to interact with it. We also present the first biological analysis of this TBTA/AmB composition, and it confirms the synergistic effects of TBTA. The report provides a detailed description of the synergism observed between a newly synthesized derivative from the group of 1,3,4-thiadiazoles (TBTA) and the antibiotic AmB, an effect that may prove to be very significant in the context of the ongoing efforts to identify new substances with antifungal properties.

## Graphical abstract

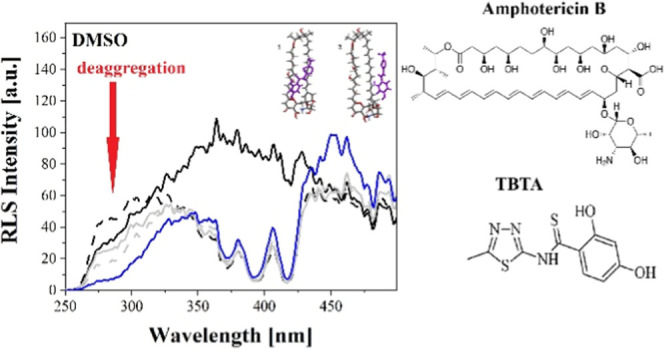

## Introduction

Amphotericin B (AmB; Scheme 1A) is a polyene antibiotic with strong antimycotic properties, first isolated from Streptomyces nodosus in 1955. However, the clinical viability of the antibiotic is limited by its high, dose-dependent toxicity. It is particularly dangerous due to its nephrotoxicity.^1^ A number of lipid formulations of amphotericin are already known that can reduce this toxicity and improve the drug’s availability. These include the AmB—ABLC lipid complex, AmB—ABCD colloidal dispersion, as well as AmB in the liposomal form—L-AmB.^2–4^ Nonetheless, despite numerous limitations, the molecule continues to be considered the “golden standard” in the treatment of various fungal infections.^5^ It has been reported in numerous scientific reports that the molecule’s toxicity is due to its aggregation in the membranes of human cells containing cholesterol,^6^ which is why monomerization of AmB before its administration would be ideal. However, according to the literature, the molecule is prone to aggregate in water media, which can significantly hinder this process. Moreover, aggregated forms of the compound show completely different spectroscopic properties compared to its monomeric forms.^7^ Conventional AmB in a form with sodium deoxycholate (D-AMB) has been used in the treatment of invasive infections, but it shows the strongest toxicity compared to other available forms of the antibiotic.^8^ Due to the surface polarization of both molecules (AmB and NaDC), their interaction leads to the formation of an associative complex, given the pharmaceutical significance of the AmB + NaDC molecular system.^1^

**Scheme 1:**
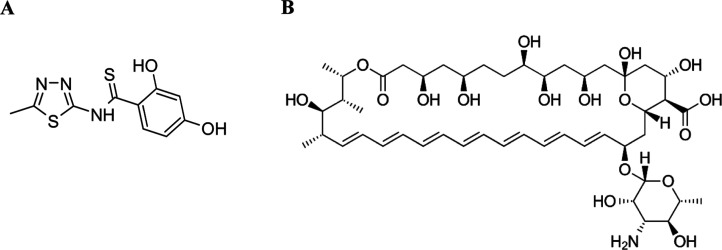
Chemical Structures of the Molecules: Panel A—TBTA and Panel B—AmB

Despite its toxicity, AmB is often used as the “last resort” drug and is still largely considered the “golden standard” in the treatment of various fungal infections,^5^ which, in the context of the coronavirus pandemic and generally excessive use of antibiotics in medicine, are becoming increasingly problematic. Synergistic effects have been discussed in the literature since the 1970s. Positive effects have been reported, for example, after combining antimycotic drugs with antibacterial agents, e.g., fluconazole and rifamycin, or AmB with tetracycline,^9^ as well as with other nonmedicinal substances such as 4-(5-methyl-1,3,4-thiadiazol-2-yl) benzene-1,3-diol.^10^ This work led to a train of thought that the best solution could be to minimize the effective dosage, which may be achievable by combining the agent with another substance. The key to identifying the most suitable system may lie in a more in-depth exploration of the mechanism of the antibiotic’s activity. In this regard, several possible theories are actively considered in the literature. The first suggests that AmB binds with ergosterol, leading to the formation of ionic channels and pores, which disrupts cellular equilibrium and results in cell death. Another is that AmB can form large, extra-membrane aggregates that kill fungal cells by extracting ergosterol from lipid bilayers.^9^

Here, we focus our efforts on exploring and explaining the mechanisms of molecular interactions taking place in a composition of AmB with TBTA (2,4-dihydroxy-*N*-(5-methyl-1,3,4-thiadiazol-2-yl)­benzothioamide), which, as we will show, has strong synergy with AmB. TBTA is a 1,3,4-thiadiazole derivative that provides a newly discovered synergistic composition, and we especially use it here to complement other recent studies that we have conducted on various 1,3,4-thiadiazole analogues.^11,12^ Our prior studies have mostly focused on the microbiological characteristics of the identified compositions, but, in this study, we seek to advance efforts to understand the key molecular mechanisms that can be associated with synergistic molecular compositions with an extensive use of spectroscopic and computational methods, which we have started to do recently with another synergistic 1,3,4-thiadiazole derivative “C1”.^13^ We include in this study more in-depth spectroscopic measurements than with C1, complementary quantum-mechanical calculations, and detailed microbiological analyses conducted specifically for TBTA. The experimental data include measurements of electronic fluorescence and absorption spectra, also using the technique of synchronic measurements, measurements of fluorescence lifetimes with the technique of single proton counting (TCSPC), measurements of fluorescence anisotropy, measurements of circular dichroism (CD), and measurements of dynamic light scattering (DLS). The computational models corroborate the hypotheses that we posed on the basis of experimental results, namely, about the possible modes of interaction between TBTA and AmB, and, furthermore, they resemble what we observed for C1.^13^ The spectroscopic and theoretical studies were complemented by preliminary microbiological analyses, which will be continued in the future, for both this and other potentially viable analogues we are currently investigating. To the best of our knowledge, this study is the first in-depth report on the molecular interactions between TBTA and the “gold standard” AmB. Especially when considered alongside studies of other 1,3,4-thiadiazole derivatives, the results provide a major advance toward a comprehensive explanation of the molecular mechanism responsible for the activity of AmB.

For the microbiological analysis that is presented, in the present study, a model biological organism, Saccharomyces cerevisiae, was chosen for the investigation of antifungal activity. Despite its numerous beneficial uses in the food industry, the organism has also been reported to be capable of pathogenic colonization. The fungus can cause throat and oral infections, which may, under extreme conditions in immunosuppressed patients, lead even to death. There are also isolated incidences, wherein they are able to change their phenotype to gain resistance to commonly used antibiotics such as fluconazole.^14^

Numerous spectroscopic studies have already been published exploring the characteristics of the AmB molecule itself, particularly in its aggregated forms.^7,15^ The absorption spectrum of the AmB molecular aggregates in water solution is relatively wide and features characteristic absorption maxima at ∼420, 385, and 360 nm as well as a very intensive band maximum at 340 nm. The electronic absorption spectrum for the monomeric form of AmB measured in, e.g., dimethyl sulfoxide (DMSO) includes sharp vibration bands at 415, 390, and 370 nm. The values of molar extinction ratios for the monomeric form are much higher than those observed in water solutions.^15^ It is also very interesting to note that despite the significant differences between the spectral profiles obtained for the monomeric and aggregated forms, they emerge in the same spectral range and the higher values of the ε ratios for the monomeric form may obscure the ones produced by the aggregated forms of AmB,^1^ which renders the electronic absorption spectra registered for the AmB molecule notoriously difficult to interpret. As follows from the literature, the electronic absorption spectra observed for AmB in different types of surfactants, e.g., NaDC micelles, are very similar to those measured in DMSO. This seems to confirm the presence of monomeric AmB in the given environment.^16^ Some researchers report that in propan-2-ol with water solutions, low order fluorescence was observed from the monomeric form, with the maximum at 560 nm, after excitation at 408 nm.^17^ Moreover, after excitation at 335 nm, strong fluorescence emission was observed with the maximum at ∼472 nm.^17,18^ With reference to the exciton splitting model, it was reported that the monomeric form shows a symmetry forbidden transition from the 2^1^Ag to the 1^1^Ag state, which explains the low efficiency of its fluorescence. In turn, the intensive fluorescence with the maximum at 472 nm (excitation at 335 nm) was associated with the allowed transition resulting from the splitting of the 1^1^Bu state of the dimeric form.^17^ Moreover, the authors also demonstrated that the degree of the exciton band split increases with the level of aggregation, which explains the absence of significant fluorescence from higher *N*-aggregates. The mentioned emission band centered at 472 nm was also assigned to oxidized forms of AmB, which was extensively described in the works by Gagoś and Czernel.^19,20^

## Materials and Methods

### Analyzed Compounds

The TBTA (Scheme 1A) derivative of 1,3,4-thiadiazoles was synthesized at the Department of Chemistry, University of Life Sciences in Lublin, as described in a previous publication.^10^ The compound was dissolved in DMSO directly before the experiment at a concentration of 1 mg/mL.

The AmB (Scheme 1B) antibiotic was purchased from Merck KGaA, ref no. Y0000005, and dissolved in DMSO. Each solution was prepared directly before the experiment and protected against exposure to light. A 1 mg/mL solution was prepared for the purposes of spectroscopic studies and a 10 mg/mL solution for the microbiological studies.

The samples were prepared at the temperature of 22 °C. The source samples were pipetted using an automatic pipet until a clear solution was obtained. The desired concentrations of the analyzed compounds were prepared directly in a quartz tray with an optical path length of 10 mm. After the compounds were dosed into the target media, the samples were stirred for approximately 2 min using the magnetic stirrer of the measurement apparatus.

### Model Organism and the Method of Growing the Fungus

The S. cerevisiae strain obtained from the collection of the University of Life Sciences in Lublin was selected for the study. In order to cultivate the biomass, yeast was grown on wort agar (BTL) or liquid YPG medium at the temperature of 30 °C. Next, fungal inoculums were prepared for the purposes of determining the antimycotic properties of the analyzed compound by dissolving the all-nigh culture in 0.9% NaCl to 1–5 × 10 ^6^ CFU/mL (relative to the isolate density standard of 0.5 on McFarland’s scale).

### Electronic Absorption, Fluorescence, and RLS Spectra

The electronic absorption spectra for TBTA, AmB, and the synergistic composition of the two compounds were measured using a UV–vis Cary 300 Bio dual beam spectrophotometer from Varian, equipped with a thermostated holder with a multicell Petri dish. During the measurement, the temperature was controlled by using a thermoelectric probe (Cary Series II from Varian) placed directly in the sample.

The excitation, fluorescence emission, and synchronic RLS spectra were measured by using a Cary Eclipse spectrofluorometer (also from Varian). All of the spectra were recorded at room temperature, at the spectral resolution of 0.5 nm. Necessary corrections were made to account for the spectral characteristics of the lamp and the photomultiplier. Measurements of synchronic (RLS) spectra were performed as described in refs 21 and^22^, with simultaneous scanning of both the excitation and emission monochromators, without intervals between the excitation and emission wavelengths, at a spectral resolution of 1.5 nm. The registered data were analyzed and prepared for publication using Grams/AI 8.0 (Thermo Electron Corporation; Waltham, Massachusetts, USE) and Origin 2023b software (USA). The emission spectra in PBS for TBTA and AmB as well as the synergistic systems were recorded with excitation at 335 nm. Excitation spectra were recorded in the emission maxima of the bands (Ex­(Em478)) nm. The emission spectra in DMSO for TBTA were recorded as the excitation at 330 nm for AmB and synergistic system at 350 nm. Excitation spectra were recorded in the emission maxima of the bands: TBTA Ex­(Em380) nm, AmB, and synergistic system Ex­(Em470) nm.

### Anisotropy Measurements

The fluorescence anisotropy in the stationary state (*r*) was calculated using polarized constituents of fluorescence with the following formula

1 $$r=\frac{I_{VV\;}-GI_{VH}}{I_{VV\;}+2GI_{VH}}$$

where *I*_VH_ is the emission intensity with vertical excitation and horizontal observation, *I*_VV_ is the emission intensity with vertical excitation and vertical observation, and *G* is the geometric coefficient correcting for the system’s polarization errors. A wire grid polarizer was used for excitation to allow UV light transmission.

### Time-Correlated Single Photon Counting

Time-correlated single photon counting (TCSPC) measurements were performed by using a FluoroCube fluorimeter (Horiba, France). The samples were excited with a pulsed NanoLED diode at 372 nm (pulse duration of 150 ps) operated at 1 MHz. To avoid pulse pile-up, the power of the pulses was adjusted to an appropriate level by using a neutral gradient filter. Fluorescence emission was recorded using a TBX-04 ps detector (IBH, JobinYvon, UK). The DataStation and DAS6 software [JobinYvon (IBH, UK)] was used for data acquisition and signal analysis. All fluorescence decays were measured in a 10 × 10 mm quartz cuvette, using an emitter cutoff filter with transmittance for wavelengths longer than 408 nm. The excitation profiles required for the deconvolution analysis were obtained without emitter filters using a light scattering cuvette. The compounds were dissolved in DMSO or PBS directly before the measurements. Each fluorescence decay was analyzed with a multiexponential model from the following equation

2 It=∑iαiexp(−tτi)

where α_i_ and τ_i_ are the pre-exponential factor and the decay time of component *i*, respectively.

Best-fit parameters were obtained through minimization of the reduced χ^2^ value as well as the residual distribution of the experimental data. The fractional contribution (*f*_*i*_) of each decay time and the average lifetime of fluorescence decay (⟨τ⟩) were calculated from the following equations

3 $$f_{i}=\frac{\alpha_{i}\tau_{i}}{\sum_{j}\alpha_{i}\tau_{i}}$$

4 $$\left\langle \tau \right\rangle =\sum_{i}f_{i}\tau_{i}$$

### Circular dichroism Measurements

CD measurements were conducted using a J-710 spectropolarimeter (Jasco) in 111–10–40 cuvettes of 1 cm path length (Hellma), at room temperature. The compounds were dissolved in PBS or DMSO directly before the measurements. The spectra of compounds dissolved in PBS were collected in the range of 250–500 at 1 nm data pitch, 100 nm/min scanning speed, 1 s response time, and 2 nm bandwidth. The spectra of compounds dissolved in DMSO were collected in the range of 250–500 at 1 nm data pitch, 50 nm/min scanning speed, 2 s response time, and 2 nm bandwidth and averaged over three acquisitions. Respective solvents were used as blanks, and their spectra were subtracted from the raw data.

### Dynamic light scattering

DLS measurements were performed with a Zetasizer Nano S analyzer (Malvern), using a 4 mW 632.8 nm laser at a scattering angle of 173°. The hydrodynamic diameter of the particles was determined from the recorded autocorrelation functions using cumulant method analysis performed with instrument software. The results were averaged over five acquisitions.

### Determination of Antimycotic Activity

The MIC value for the TBTA compound was measured using the microdilution method described in detail in CLSI M27-A2 ^23^ for yeast. MIC corresponds to the lowest concentration of an antifungal agent causing a reduction in visible turbidity compared with the control sample. The activity is determined in accordance with the criteria proposed by Morales^23^ as string or good for MIC <100 μg/mL, moderate for MIC: 100–500 μg/mL, and lack of activity, inactive compound for MIC: >1000 μg/mL. The tests were performed for 9 TBTA concentrations—256, 128, 64, 32, 16, 8, 4, 1, and 0.5 μg/mL—and 8 AmB concentrations—4, 2, 1, 0.5, 0.25, 0.125, 0.06, and 0.03 μg/mL, prepared on Roswell Park Memorial Institute medium (RPMI-1640). The method allowed us to arrive at a matrix of TBA and AmB concentrations. TBTA and the antibiotic were applied onto a sterile microculture plate at 100 μL/cell, and the fungal inoculum at 50 μL. S. cerevisiae growth control (containing none of the analyzed compounds) and background control (containing only the growth medium) samples were also prepared on the plate. The experiment was performed in triplicate, and the obtained results were averaged accounting for the background control. The optical density of the fungal culture (OD600) was measured for 48 h at 28 °C using an automated microorganism growth reader (system Bioscreen C, Labsystem, Helsinki, Finland) at the wavelength of 600 nm, with automatic registration once every 2 h for each cell. Turbidimetric growth curves were obtained depending on changes in the fungal growth OD600 values obtained for each concentration of the antifungal agent and for the control. The growth curve parameters (i.e., maximum specific growth rate, lag time, and doubling time) were determined using the PYTHON script by Hoeflinger et al.^24^ The value of MIC^100^ was taken as the lowest concentration, causing 100% inhibition of fungal growth after 48 h. In accordance with the recommendations of the American Society for Microbiology,^25^ the fractional inhibitory concentration index FIC was calculated from the following equation: ∑FIC = FIC_AmB_ + FIC_TBTA_, where FIC_AmB_ = MIC_AmB_ in the presence of TBTA/MIC_AmB_ individually, and by analogy FIC_TBTA_ = MIC_TBTA_ in the presence of AmB/MIC_TBTA_ individually. The following interaction criteria were employed: ∑FIC ≤0.5—synergy, ∑FIC >0.5 do ≤ 1—additivity, ∑FIC >1 to <2—antagonistic effect, ∑FIC ≥2—antagonism.

### Computational Searches for Stable Structural Motifs

An automated search scheme was used to search for stable structural motifs of one TBTA molecule binding to one AmB molecule in solution. The general approach was to generate a pseudoexhaustive set of possible AmB/TBTA geometries and then optimize every one with the GFN2-xTB^26^ semiempirical method; the GFN2-xTB method is run within the DFTB + program^27^ and used with a GBSA implicit solvation model.^28^ The AmB structural space is simplified by using only one AmB reference geometry to generate all the potential structures, this reference geometry was obtained by running the CREST program^29^ on a reasonable starting geometry (with the default implicit water solvation model included). To generate the structures, a set of evenly spaced grid points is first generated around the reference AmB structure, wherein the grid points were taken from two superimposed spherical grids generated from two atoms on opposite sides of the AmB macrocycle. The long axes of the AmB and TBTA monomers were then oriented along the *x*-axis to define standard orientations for each, and then, the TBTA molecule center was placed at each grid point. The “head-to-head” (HtoH) searches correspond with the case where TBTA’s standard orientation is unchanged vs the AmB reference orientation, while the “head-to-tail” (HtoT) searches correspond with the case where TBTA’s standard orientation is rotated around the *y*-axis by 180°. For every considered grid-point placement, distinct geometries were created by incrementally sampling possible TBTA rotations. This was done by first rotating TBTA around the *x* axis in increments of 60° and then applying a tandem pair of rotations around the *y*-axis (either 0° or 30°) and around the *x*-axis (in increments of 90°); the tandem rotations allow the molecule to precess (by 30°) around the *x*-axis. Every generated geometry is then fully optimized at the (implicitly solvated) GFN2-xTB level of theory.

## Results and Discussion

### Electronic Fluorescence and Absorption Spectroscopy

First, Figure 1 presents the electronic absorption spectra registered for the studied molecules: TBTA, AmB, and the synergistic composition of TBTA + AmB. Panel A shows the absorption spectra for the molecules in DMSO. For TBTA, we observed a wide absorption band with the maximum at ∼290 nm which, according to literature, corresponds to the π→π* electronic transition taking place in the molecule’s chromophoric system.^30^ The TBTA spectrum in PBS was fairly wide on the long-wave side, reaching ∼350 nm and peaking at ∼280 nm for the main absorption band. As follows from Kasha’s exciton splitting theory, the band’s enhancement should be associated with the possible presence of aggregated TBTA molecules. Next, the broken black line indicates the AmB spectrum characteristic of its monomeric form, as described in the literature.^17^ Furthermore, the absorption spectra obtained for the TBTA + AmB complex are presented as measured for several TBTA to AmB ratios/concentrations. As we can observe, the gray line (synergistic system) practically overlaps with the spectra of the respective compounds in the given solvent.

**Figure 1:**
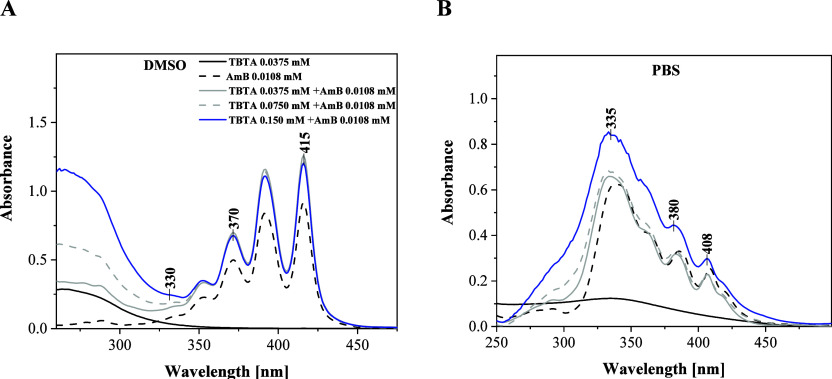
Electronic absorption spectra for TBTA, AmB, and the synergistic composition of TBTA + AmB, in DMSO—Panel A; Panel B—electronic absorption spectra for analogous systems in the PBS buffer medium.

Panel B of Figure 1 shows results analogous to panel A but obtained in PBS, i.e., the medium where the aggregation effects are the most pronounced for AmB molecules.^31^ Here, we can observe a significant impact of aggregation effects, both in terms of TBTA and AmB, as well as the relevant composition showing synergistic properties in biological studies. In the spectrum of TBTA itself, the main absorption band maximum was shifted from ∼280 nm (in DMSO) to ∼335 nm; we can also observe clear enhancement on the longwave side, reaching as far as ∼450 nm. In this case, the region should be associated primarily with the formation of head to tail dimer systems but also larger aggregated structures. The black broken line represents the absorption spectrum for AmB, typical of the compound’s aggregated forms, preferred in this type of solutions.^31^ The 415 nm maximum observed in DMSO was shifted to 408 nm, while on the short-wave side of the spectrum, we can observe the emergence of a wide band peaking at ∼335 nm. Based on well-established literature data as well as our prior studies, these effects reflect the formation of both head to tail and card pack types of aggregates.^32^ Next, the spectrum registered for the synergistic composition (gray line, gray broken line, and blue line) also showed a maximum at ∼335 nm. Interestingly, mathematical addition or subtraction of the spectra collected for TBTA, AmB, and the composition yielded results almost exactly matching those of the other spectra. Hence, in the case of the buffer solution, we could much more clearly see the impact of aggregation effects. For the first two volumes of TBTA added to the solution with AmB, we observed only a slight band increase in the aggregate region. Only with the last addition was the absorption band increased in this region but with a simultaneous significant increase of the band characteristic of the AmB monomer. It can be preliminarily suggested that the addition of TBTA facilitates the disaggregation of AmB in solutions such as PBS. The mechanism of this effect on the AmB molecule has already been described for multiple compounds, and it has been confirmed that it does have a bearing on AmB’s toxicity. Analogous spectra for lower concentrations with absorbance under 0.1 are presented in the SM, as shown in Figure S3. The shapes of the spectra are consistent with those presented above, which shows that the discussed synergistic effect is not influenced by effects related to reabsorption. Additionally in the SM, Figure SM4 presents the concentration dependence of the absorbance ratio at 335 (aggregated form) and 408 nm (monomeric form) in the absorption spectrum at the synergistic composition of TBTA + AmB in PBS, in which we can see that the addition of 0.150 mM TBTA causes deaggregation of the synergistic system.^33^

### Fluorescence Spectroscopy—Measurements of Fluorescence Emission Spectra and Fluorescence Anisotropy

To complement the measurements of the electronic absorption spectra, fluorescence emission spectra were also measured, as shown in Figure 2. The presented results were obtained for TBTA, AmB, and the synergistic composition in PBS buffer solution. In the supplementary part (Figure SM1), we also provided fluorescence emission spectra registered in DMSO, corresponding to the absorption spectra shown in Figure 1A. In the case of the AmB molecule, fluorescence spectroscopy plays a significant role in uncovering the mechanisms of its aggregation, both in organic solvents and water solutions. A structure of oscillative states was observed in the AmB emission spectrum in water solutions which, based on the exciton splitting theory, was associated with dimeric forms of the AmB molecule.^34,35^ We also know that fluorescence emission occurs in AmB from a different state than that to which the molecule is originally excited.^36^ Due to the aforementioned symmetry principles, in the case of AmB, the quantum efficiency of fluorescence is approximately 2 orders of magnitude higher in the dimeric form, as compared to the corresponding monomeric or aggregated forms.^37^ The spectra were registered in a PBS buffer system to allow a better look at the effects related to aggregation. The fluorescence emission spectrum obtained for TBTA after excitation at the maximum of the absorption spectrum peaked at ∼335 nm and was typical of the S_1_→S_0_ transition in the enol form of the compound (Scheme 1A). In turn, the AmB molecule in water systems, when excited at the maximum of the band associated with the dimerized forms of the compound, produced a characteristic wide band with the maximum at ∼478 nm.^38,39^ Once a suitable amount of TBTA was added, we arrived at the spectrum of the synergistic composition. However, its shape was somewhat irregular, which likely resulted from the weak emission observed for TBTA itself. Nonetheless, a very interesting effect was observed where the obtained emission spectrum departed from the spectrum characteristic of AmB and was shifted toward the shape characteristic of TBTA. Figure SM2 in the SM presents the excitation spectra registered for the relevant systems corresponding to the above emission spectra.

**Figure 2:**
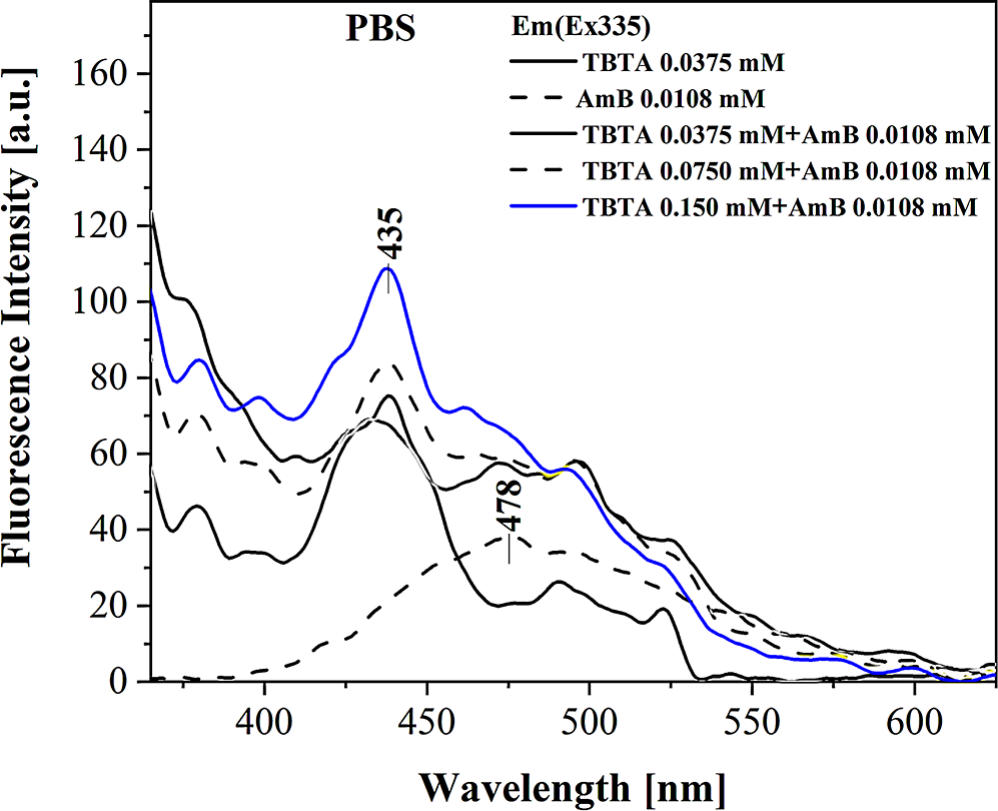
Example fluorescence emission spectra for TBTA, AmB, and their synergistic composition in buffer PBS medium, analogous to the spectra in Figure 1.

The changes observed in the fluorescence emission spectra and the absorption spectra presented in Figure 1 suggest the existence of a molecular mechanism responsible for the activity of the analyzed composition that revealed fairly significant synergism in microbiological studies. Based on the results obtained from the spectroscopic measurements of electron emission and absorption, one might preliminarily conclude that TBTA triggers breakup of AmB dimers described in the literature.^40^ Due to the disaggregation of AmB, the spectrum characteristic of the said dimers decayed, and we mainly observed emission from TBTA molecules, which is significantly stronger than that from AmB in the given medium. This outcome is not new to the literature and has already been reported, e.g., for sodium deoxycholate and AmB in micellar systems.^40^

Next, by way of complementing the already discussed results, measurements of fluorescence anisotropy were conducted (examples are shown in Figure 3). The preliminary observation made on the basis of the electronic fluorescence and absorption spectra (Figures 1 and 2) pointed to disaggregation taking place in the TBTA + AmB system. Hence, we decided to check whether the system’s anisotropy changed relative to AmB. The measurements were conducted for AmB and TBTA combined with AmB, in PBS buffer. The anisotropy value obtained for AmB and TBTA (separately) was ∼0.08, whereas the result observed for the thiadiazole + AmB composition showed a slight decrease already at a wavelength of ∼470 nm. Anisotropy measured for the composition was ∼0.035. It is noteworthy that while the values of fluorescence anisotropy are undeniably low, they show a clear decreasing trend, which corroborates the preliminary hypothesis regarding the disaggregation of AmB dimers under the influence of TBTA molecules.

**Figure 3:**
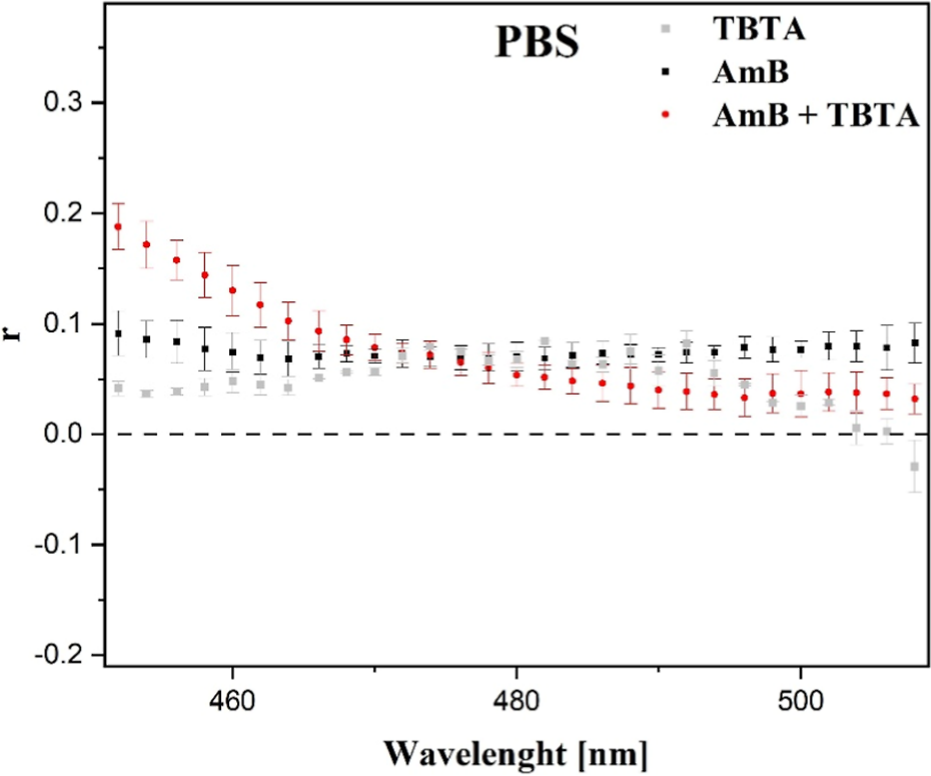
Example fluorescence anisotropy curves for AmB and the synergistic composition in a buffer PBS medium.

#### Analysis of circular dichroism Spectra—CD

Further exploration of the observed changes in the balance between the aggregated and monomeric forms in the synergistic composition involved measurements of CD spectra for AmB in systems similar to those shown in Figures 1–3. The results are listed in Figure 4. The AmB molecule is highly chiral and produces a very clear CD spectrum (black solid line in Figure 4). We could clearly observe both positive and negative Cotton effect in the presented spectra with peaks at 325 and 350 nm, respectively. The shape of the spectrum agrees with those already described in the literature.^41^ This evidences the correct rotation of the light polarization plane by the AmB solution. Thus, reduction of both the CD minimum and the maximum suggests greater rotational and conformational freedoms of AmB molecules, consistent with disaggregation. Titrating AmB with the TBTA thiadiazole, analogous to Figure 1, allowed us to observe such a decrease in the intensity of AmB CD spectra, clearly pointing to ongoing dissociation of AmB dimers. In DMSO solution, AmB is less prone to aggregation, so its molecules are not trapped in an asymmetric environment, which results in a less pronounced signal CD spectra that are able to reflect considerably better the processes involved in disaggregation of AmB aggregates compared to absorption spectra (as well as, to a lesser extent, fluorescence emission spectra) due to the specific chiral properties of the AmB molecule itself, especially its aggregated forms. As such, the CD spectra provided us with more direct insight into the process of the antibiotic’s disaggregation.

**Figure 4:**
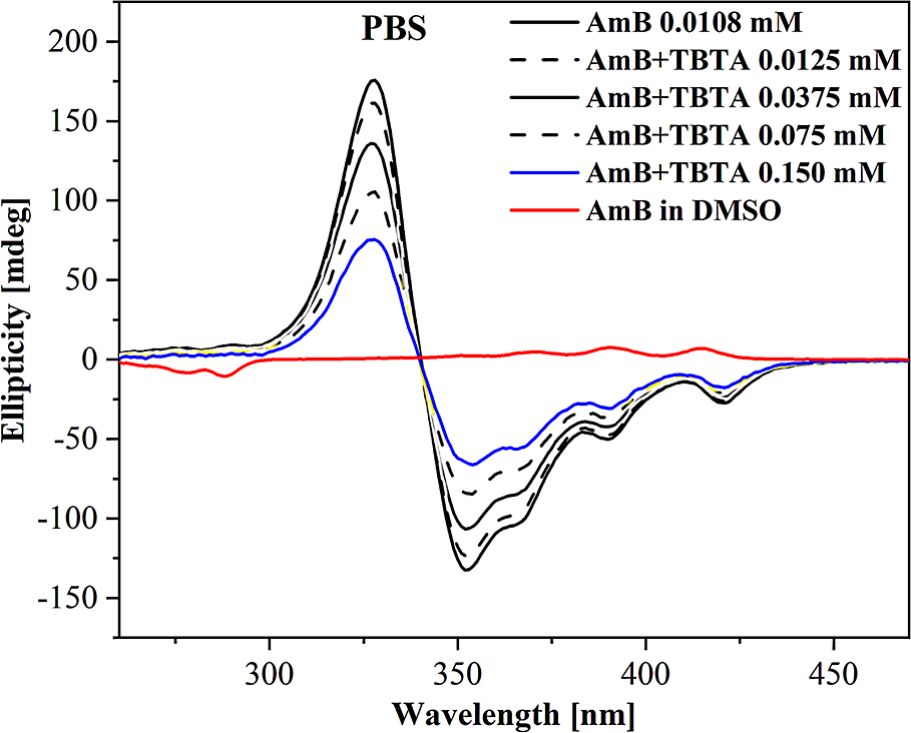
CD spectra for: TBTA, AmB, and the synergistic TBTA + AmB composition, in PBS.

### Analysis of Resonance Light Scattering Spectra (RLS)

With a view to more closely analyzing the impact of increasing TBTA content relative to AmB, RLS spectra were recorded to complement the CD spectroscopy data. Under the theory proposed by Pasternack and Collings in 1993,^42^ RLS spectra can confirm the presence or absence of aggregated forms in a given system, with the intensity of the RLS signal increasing exponentially with the number of aggregates (dimers) present. This means that the method can provide very illustrative data in studies of this type. As follows from theory, RLS spectra should be located in the vicinity of the absorption spectra from aggregated forms associated with the intramolecular transit to lower exciton levels.

Notably, measurements of synchronic spectra are able to clearly reflect disaggregation in more complex molecular systems such as TBTA, AmB, and combinations of the two. Given our already described assumptions regarding interactions between thiadiazole and AmB molecules, we decided to measure synchronic RLS spectra in the hope of further corroborating out research hypotheses. Both panels of Figure 5 show RLS spectra recorded for systems analogous to those presented in Figure 1, panels A and B, and observable spectral changes are evident. In Panel A, we can see, for the analyzed compositions of TBTA + AmB in DMSO, that with the increasing content of TBTA in the composition, the intensity of the RLS spectra visibly decreases. A caveat, however, is that the intensity of RLS spectra initially increased after the first addition of TBTA, but after subsequent additions, the RLS spectral region between 150 and 350 nm started to rapidly lose intensity. Above a wavelength of 350 nm, only very slight changes in spectral intensity were registered. In the synergistic system with the highest content of TBTA (0.149 mM), the RLS spectrum was observed to gain intensity above 430 nm, but the increase was not particularly significant. The effect is even more apparent in Panel B, and the general pattern of spectral behavior in respective regions was fairly analogous to that in Panel A. In both panels, we can also note the oscillative character of the obtained spectra, which may evidence the presence of aggregated forms of various sizes in the system, i.e., dimers and larger *N*-aggregates.^17^ Panel B clearly illustrates a significant decrease in the intensity of RLS spectra already after the second addition of TBTA, within the spectral range from 150 to ∼320 nm. With further additions of TBTA into the combination, the intensity of the RLS spectra continued to rapidly deteriorate in the first of the regions mentioned above. It should be noted at this point that the final decrease in the intensity of the RLS spectra was observed in a very particular spectral region from 250 to ∼430 nm. This may provide evidence of the breakup of aggregated forms of a specific size. Once TBTA molecules start to interact with AmB molecules, systems of specific size are formed, for which the RLS signal above 420–430 nm remains at a specific, stable level.

**Figure 5:**
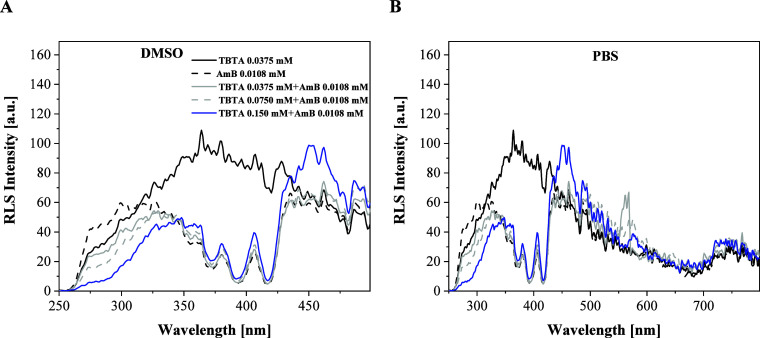
RLS spectra for: TBTA, AmB, and the synergistic composition of TBTA + AmB, in DMSO—Panel A; Panel B—RLS spectra recorded for the same systems in the buffer PBS medium.

The effect described above was visible even to the “naked eye”, as evident in Figure 6, showing a cuvette containing a sample of the TBTA + AmB composition, which, after excitation with UV light (365 nm), produced clearly more opalescent light compared to AmB or TBTA itself. This fact further corroborates the hypothesized disaggregation of AmB structures in the presence of TBTA.

**Figure 6:**
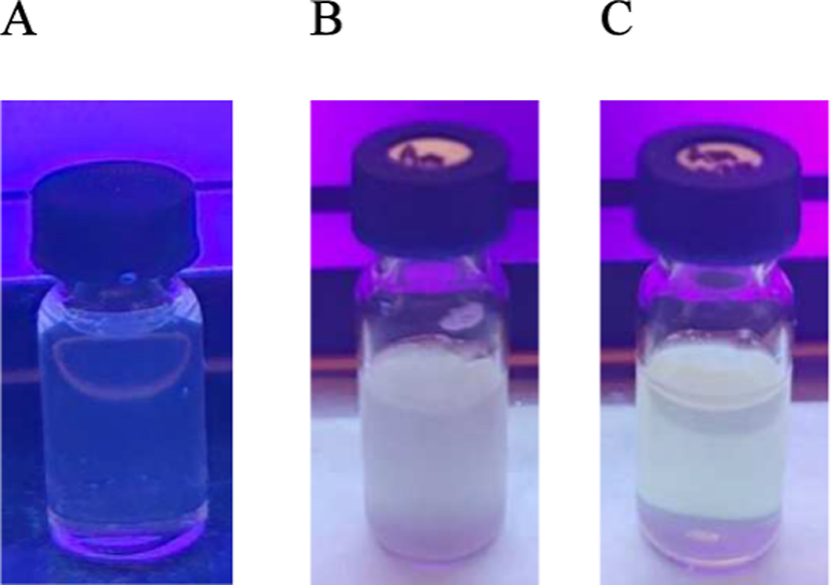
TBTA 0.0750 mM sample (A), AmB 0.0108 mM sample (B), and TBTA + AmB 0.0750 mM + 0.0108 mM sample (C) excited with 365 nm UV light in the buffer PBS medium.

### Fluorescence Spectroscopy—Fluorescence Lifetimes

In the next step, the response of the analyzed systems was analyzed by using time-resolved measurements. For systems analogous to those presented in Figure 1, fluorescence lifetimes were measured using the method of time-correlated single photon counting (TCSPC). After pulse excitation with the use of a 372 nm diode, fluorescence intensity was measured over time with a 408 nm cut-on filter. Panel A in Figure 7 shows fluorescence decays obtained for DMSO solution of AmB, where monomeric forms of AmB prevail, while the decays in Panel B were obtained in PBS solution, wherein AmB’s aggregated forms are dominant. The decays were described by a three-exponential model, from which component lifetimes and their respective fractions were calculated (Table 1), and illustrate the dynamics of said lifetimes in DMSO and PBS, respectively. Two-exponential models did not fit the decays well enough, while including a fourth component did not improve the fits significantly. The mean fluorescence lifetime measured in DMSO was ∼1.71 ns, while for the synergistic composition, it changed to ∼2.14 ns. As such, the changes were not significant in DMSO, which generally confirms the assumption that in this medium, we were dealing primarily with monomeric forms of the compounds or the composition thereof. The decay profile obtained from this experiment was also relatively stable. Mean lifetimes increased slightly with the addition of TBTA, while the values of the shortest constituent, whose contribution to the decay was by far the largest, decreased. We therefore observed slight lengthening of the mean lifetime after the addition of TBTA. In the PBS system, the mean lifetime measured for AmB was 1.61 ns, while in the case of the synergistic composition, it was noticeably extended up to ∼2.04 ns. As can be seen in Table 1, the value of the shortest constituent increased slightly with the addition of TBTA, but its contribution decreased 6-fold, while that of the longer constituent sharply increased. In the PBS system, i.e., a medium far more conducive to aggregation than DMSO, the decay values point to the process of the system’s disaggregation with the addition of the thiadiazole, which aligns with the results and observations presented above. TBTA itself gives rise to a very weak fluorescence signal under conditions used in the measurement. Fluorescence decays of TBTA alone are shown in.

**Figure 7:**
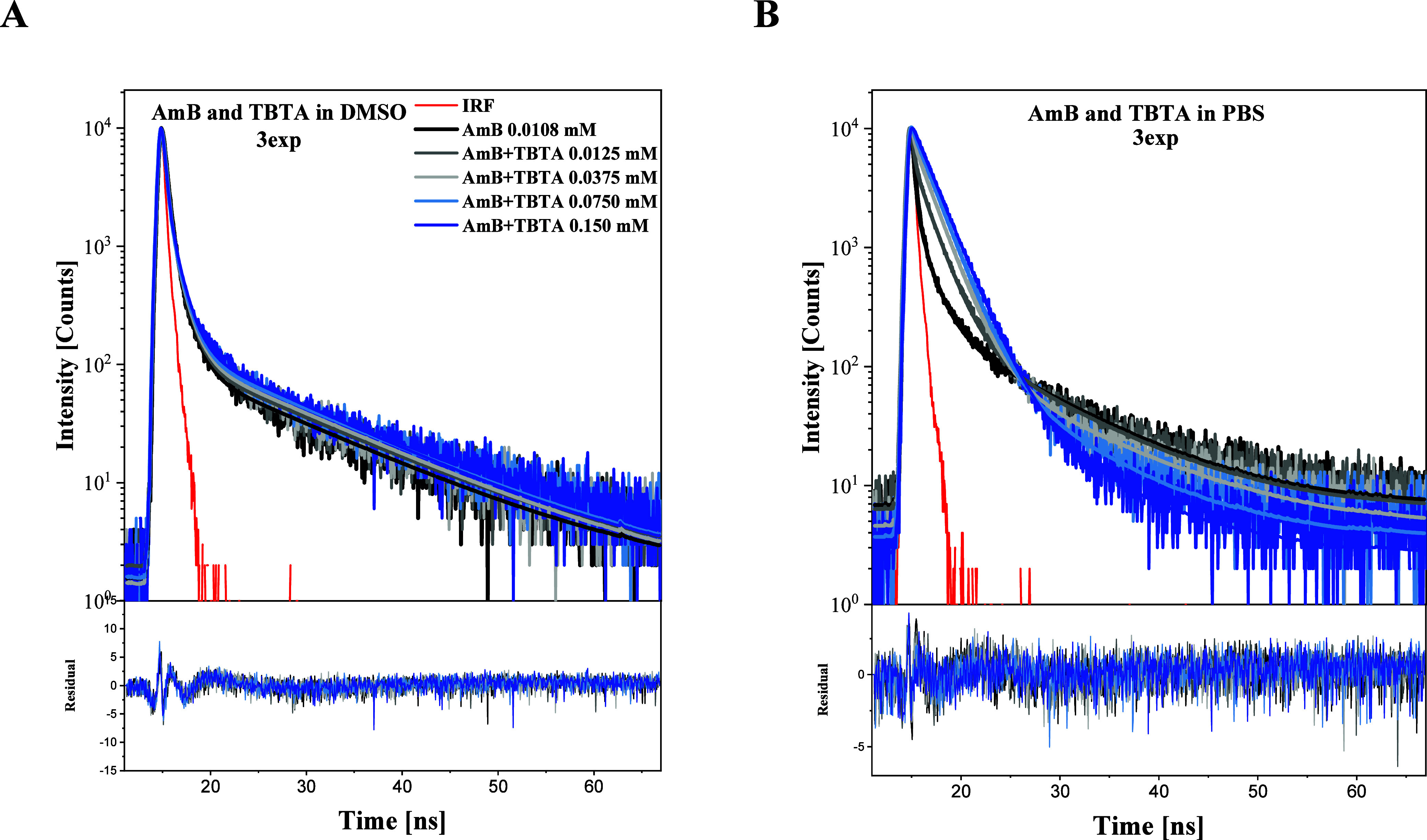
Typical fluorescence decay profiles for AmB and the synergistic composition TBTA + AmB, in DMSO (Panel A) and PBS (Panel B). Upper panels show the decays and respective 3-exponential fits. Lower panels show the residual distribution.

**Table 1:** Values of Fluorescence Lifetime (τ in ns) and Fractional Intensity (*f*), with Their Corresponding Standard Deviations, Measured for AmB Alone and in the Presence of TBTA^a^ ^,^ ^b^

	probes, mM	⟨τ⟩, ns	τ1, ns	τ2, ns	τ3, ns	*f*1	*f*2	*f*3	χ^2^
DMSO	TBTA0.0125	1.05 ± 0.03	0.11 ± 0.01	1.29 ± 0.02	6.3 ± 0.1	0.620 ± 0.001	0.281 ± 0.002	0.099 ± 0.004	1.54
	AmB0.0072	1.71 ± 0.06	0.22 ± 0.01	1.19 ± 0.03	12.0 ± 0.3	0.700 ± 0.001	0.189 ± 0.002	0.111 ± 0.005	1.61
	AmB + TBTA0.0072 + 0.0125	1.84 ± 0.05	0.23 ± 0.01	1.25 ± 0.04	12.2 ± 0.2	0.699 ± 0.001	0.181 ± 0.002	0.120 ± 0.003	1.72
	AmB + TBTA0.0072 + 0.0019	1.91 ± 0.06	0.21 ± 0.01	1.21 ± 0.03	12.2 ± 0.2	0.678 ± 0.001	0.196 ± 0.002	0.126 ± 0.004	1.71
	AmB + TBTA0.0072 + 0.0375	2.09 ± 0.05	0.20 ± 0.01	1.27 ± 0.03	12.7 ± 0.2	0.662 ± 0.001	0.205 ± 0.002	0.134 ± 0.003	1.73
	AmB + TBTA0.0072 + 0.0750	2.14 ± 0.05	0.19 ± 0.01	1.27 ± 0.03	12.5 ± 0.2	0.654 ± 0.001	0.206 ± 0.002	0.140 ± 0.004	1.75
PBS	TBTA0.010	1.71 ± 0.02	0.057 ± 0.001	1.74 ± 0.01	7.2 ± 0.1	0.319 ± 0.001	0.588 ± 0.001	0.093 ± 0.002	1.39
	AmB0.00108	1.61 ± 0.09	0.07 ± 0.01	1.50 ± 0.04	9.0 ± 0.2	0.685 ± 0.001	0.171 ± 0.002	0.144 ± 0.009	1.34
	AmB + TBTA0.00108 + 0.010	1.76 ± 0.04	0.07 ± 0.01	1.75 ± 0.02	9.1 ± 0.2	0.429 ± 0.001	0.471 ± 0.002	0.100 ± 0.004	1.39
	AmB + TBTA0.00108 + 0.0149	1.93 ± 0.02	0.10 ± 0.01	1.87 ± 0.01	9.7 ± 0.2	0.246 ± 0.001	0.690 ± 0.001	0.064 ± 0.002	1.24
	AmB + TBTA0.0108 + 0.0375	1.90 ± 0.02	0.10 ± 0.02	1.92 ± 0.01	7.9 ± 0.2	0.174 ± 0.002	0.776 ± 0.002	0.050 ± 0.002	1.34
	AmB + TBTA 0.00108 + 0.0750	2.04 ± 0.02	0.17 ± 0.01	2.07 ± 0.01	7.3 ± 0.3	0.111 ± 0.001	0.855 ± 0.001	0.033 ± 0.002	1.28

a Measurements were performed using a filter with cut-on 408 nm at the excitation wavelength of 372 nm. Cf.

b DLS measurements.

To complement, sum up, and further corroborate the conclusions following from the above experiments, the final stage of the spectroscopic study entailed measurements using the technique of DLS. The DLS determinations allowed us to measure changes in the hydrodynamic radii of AmB in the form of aggregates and the possible TBTA adducts formed in the synergistic composition. As follows from the obtained results, the mean hydrodynamic diameters measured for the synergistic composition were always lower than the values obtained for known AmB aggregates alone (Figure 8 and Table 2). The mean hydrodynamic diameters for the aforesaid systems were, respectively, 874 and 825 nm for AmB in PBS and DMSO. Meanwhile, for the synergistic composition, the values were decidedly lower, 550 and 345 in PBS and DMSO, respectively. This observation clearly evidences the fact that the structures of AmB particles were significantly reduced in size. The particle size is also more uniformly distributed in the AmB/TBTA mixture than in either of these compounds alone (Figure 7B).

**Figure 8:**
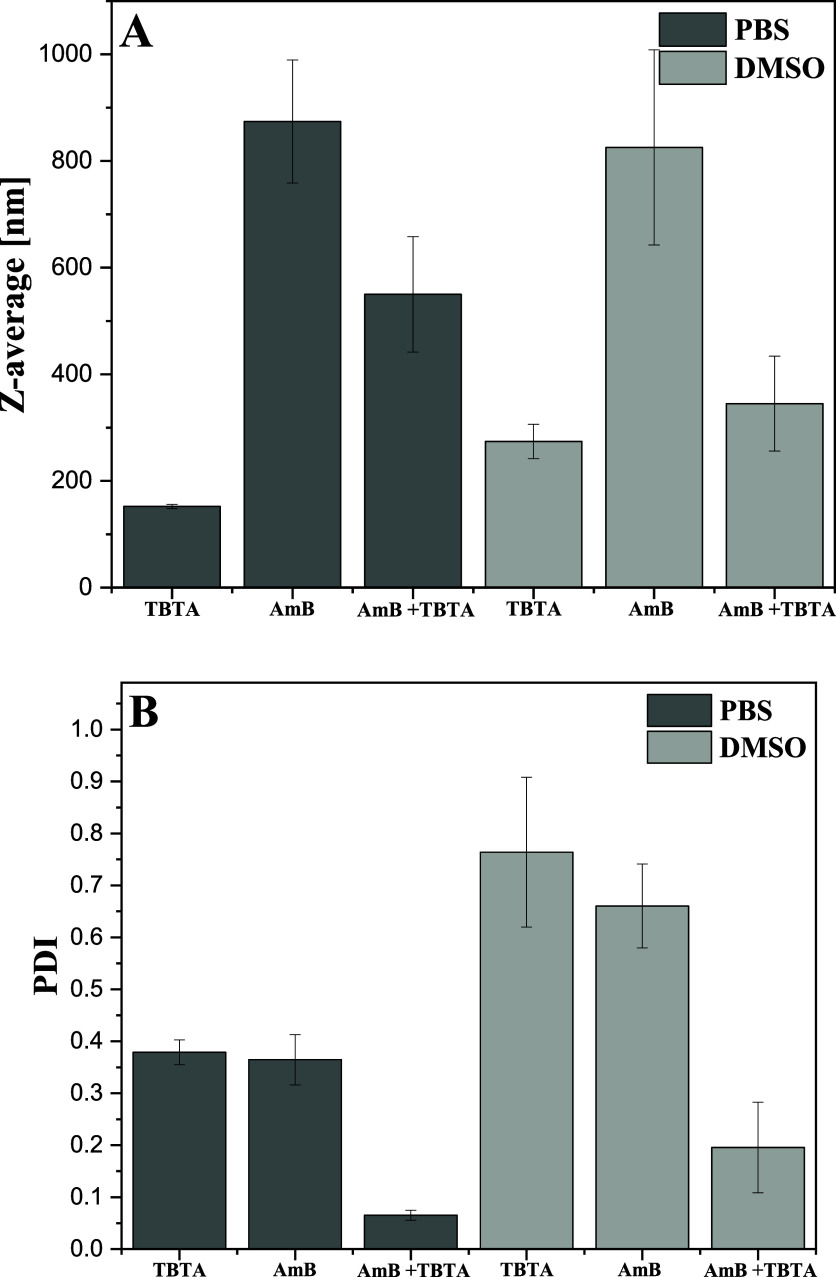
Mean hydrodynamic sizes (Panel A) and polydispersity indices (Panel B) for AmB particles alone and in the synergistic system of AmB + TBTA, in PBS or DMSO at 22 °C, measured using the technique of DLS, with their corresponding standard deviations.

**Table 2:** Average Molecule Sizes and Standard Deviations for AmB, TBTA, and the Synergistic Composition Measured in PBS and DMSO Using the DLS Method^a^ ^,^ ^b^

	Z-average		PDI	
probes	mean [nm]	sd [nm]	mean	sd
TBTA in PBS	152	4	0.07	0.01
AmB in PBS	874	115	0.38	0.02
AmB + TBTA in PBS	550	108	0.36	0.05
TBTA in DMSO	274	32	0.20	0.09
AmB in DMSO	825	183	0.76	0.14
AmB + TBTA in DMSO	345	89	0.66	0.08

a The presented values show means and standard deviations from at least five independent measurements.

b Amb 0.0108 mM, TBTA 0.0150 mM.

### Computational Models of TBTA Interactions with AmB in Solution

Computational models were used to assess how a TBTA molecule interacts most favorably with an AmB molecule in solution. Figure 9 presents the binding energies (Δ*E*′s) of the most stable TBTA/AmB dimer geometries that were found at the (implicitly solvated) GFN2-xTB level of theory; the Δ*E* values are computed from total GFN2-xTB energies, so they do not include any vibrational corrections, and they are referenced to the most stable TBTA geometry that was found at this level theory, TBTA (a), as shown in Figure 9A. The TBTA (b) geometry was also considered because its energy fell within 1 kcal/mol of TBTA­(a) at this level of theory, and it is obtained from TBTA (a) by rotating the terminal free hydroxyl group. HtoH (head-to-head) and HtoT (head-to-tail) orientations of TBTA were considered separately for each geometry, resulting in the four separate data sets shown in Figure 9A. In the case of HtoH, the terminal hydroxyl group of TBTA is oriented in the same direction as the mycosamine group of AmB. Two geometries, **1** and **2,** as shown in Figure 9, were found to be particularly stable compared to the rest of the generated structures, and their geometries are shown in Figure 9B. In both cases, they correspond with HtoH orientations, wherein the terminal hydroxyl group of TBTA creates an intermolecular hydrogen bond with an exposed -*O*- group of AmB, either from the mycosamine ring in the case of **2** or from the AmB macrocycle in the case of **1**. Generally speaking, this shows that the preferred binding motifs of TBTA resemble what we observed recently for the C1 thiadiazole,^13^ which is encouraging because both thiadiazoles were found to synergize favorably with AmB.

**Figure 9:**
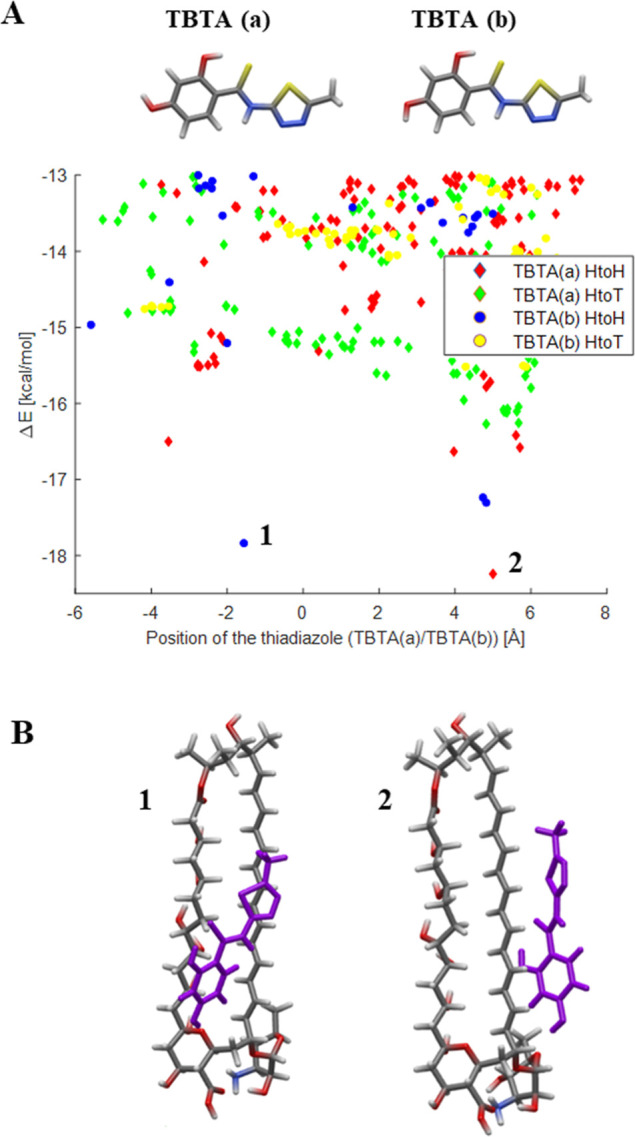
A) Computed binding energies, Δ*E*_bind_, of all the AmB/TBTA dimers that were generated; Δ*E*_bind_ is computed at the GFN2-xTB level of theory as the difference in energy between the optimized dimer and each of the optimized reagents. The HtoH (“head-to-head”) data sets refer to geometries where the terminal hydroxyl group of TBTA is positioned “down” relative to the reference AmB geometry (see panel B), whereas the HtoT (“head-to-tail”) data sets refer to geometries where the C1 hydroxyl group is initially positioned “up”. Each Δ*E* value is plotted vs the y coordinate TBTA’s central N atom, such that a negative value indicates TBTA is positioned near the polyol chain and a positive value implies it is near the polyene chain. (B) Two most stable TBTA/AmB dimers are shown as **1** and **2**.

Although hydrogen bonds are one of the easiest-to-identify contributors to the overall structure stability of the dimers, a plethora of comparable noncovalent interactions occur as well in the stable dimer structures, such as C–H···O types of hydrogen bonds or π- π stacking interactions between the thiadiazole core and the conjugated double-bond network along the polyene chain. These interactions arguably best characterize **1** and **2**, as they are seen to use these interactions to interact with the hydrophobic parts of AmB. The TBTA molecule in **2** interacts clearly with AmB via π- π stacking interactions with the polyene chain, while TBTA in **1** engages mostly in dispersion-assisted C–H···X interactions (where X is an atom from TBTA) within a hydrophobic pocket of AmB that straddles the polyene and polyol chains. As a final point, we note that in the absence of the solvation model, the stable structures at this level of theory will instead tend to engage the polar regions of AmB in order to maximize the number of intermolecular hydrogen bonds (most often creating at least one hydrogen bond with the polyol chain and another with the mycosamine group). This also points out a word of caution that electron correlation and solvation effects are very important in these systems, but ultimately the models confirm that it is the propensity of TBTA to engage the hydrophobic regions of AmB that distinguish its ability to interact synergistically with it, while also forming an intermolecular hydrogen bond with an hydrogen bond acceptor of AmB that is relatively sheltered from solution. The fact that these two binding sites are spatially mutually exclusive from one another also hints at why a higher TBTA concentration is needed in order to “block” other incoming AmB molecules, although further explicitly solvated modeling efforts on larger aggregates are needed to investigate this further.

### Antifungal Activity of the Studied Compositions

To confirm the antimycotic properties of TBTA and AmB, a preliminary microbiological analysis was performed. To this end, a microdilution checkerboard essay was performed, which allowed us to determine the effective inhibitive concentration of the synergistic system. Sachcharomyces cereviasiae was selected as the model fungal organism. It is a common choice in studies on antifungal activity and antibiotic resistance due to the fact that it is the best studied eukaryotic organism.^43,44^ The preliminary biological study confirmed the synergistic interaction between TBTA and AmB. Figure 10 shows example regression curves for S. cerevisiae on the RPMI- 1640 medium. Panel A presents the control sample. Panel B illustrates the growth of the fungi after the administration of TBTA concentrated below the MIC value (64 μg/mL). Panels C, E, and G preset yeast growth under the influence of AmB concentrated below the MIC value. As shown in biological studies, the BIC value for AmB itself is 1 μg/mL. Notably, in the case of AmB, any reduction of the dosage that simultaneously does not hinder its therapeutic effect would be a biologically beneficial result. Meanwhile, as seen in Panel D of Figure 10, the addition of merely 8 μg/mL of TBTA to AmB concentrated at 0.5 μg/mL resulted in inhibition of the fungal growth. With the addition of 16 μg/mL of TBTA, fungal growth was inhibited at as low 0.25 μg/mL AmB (panel F), i.e., a dose four times lower than required in the case of AmB alone. The MIC value for TBTA on its own was 128 μg/mL, indicating poor antimycotic activity as such. At the same time, however, one should note the growth lag time for S. cerevisia which doubled under the influence of TBTA—Panel B, while the doubling time increased from 21.1 to 35.9 h. This effect may signify the capacity to slow yeast growth, which in turn may translate to a lower MIC for AmB in the synergistic system. Table 3 presents the values of ΣFIC for the AmB and TBTA systems that inhibited the growth of S. cerevisiae. It is clear that the composition of the antibiotic concentrated at 0.25 μg/mL and TBTA at 16 μg/mL revealed strong synergism. The other two composition systems showed additive activity. The result registered for AmB dosed 8 times below its MIC when mixed with 64 μg/mL of TBTA was still very positive biologically, even if the standards of the American Society for Microbiology would classify it as an additive, rather than synergistic effect. Nonetheless, the potential ability to extensively reduce the effective dose of a highly toxic drug such as AmB can prove invaluable in clinical applications.

**Figure 10:**
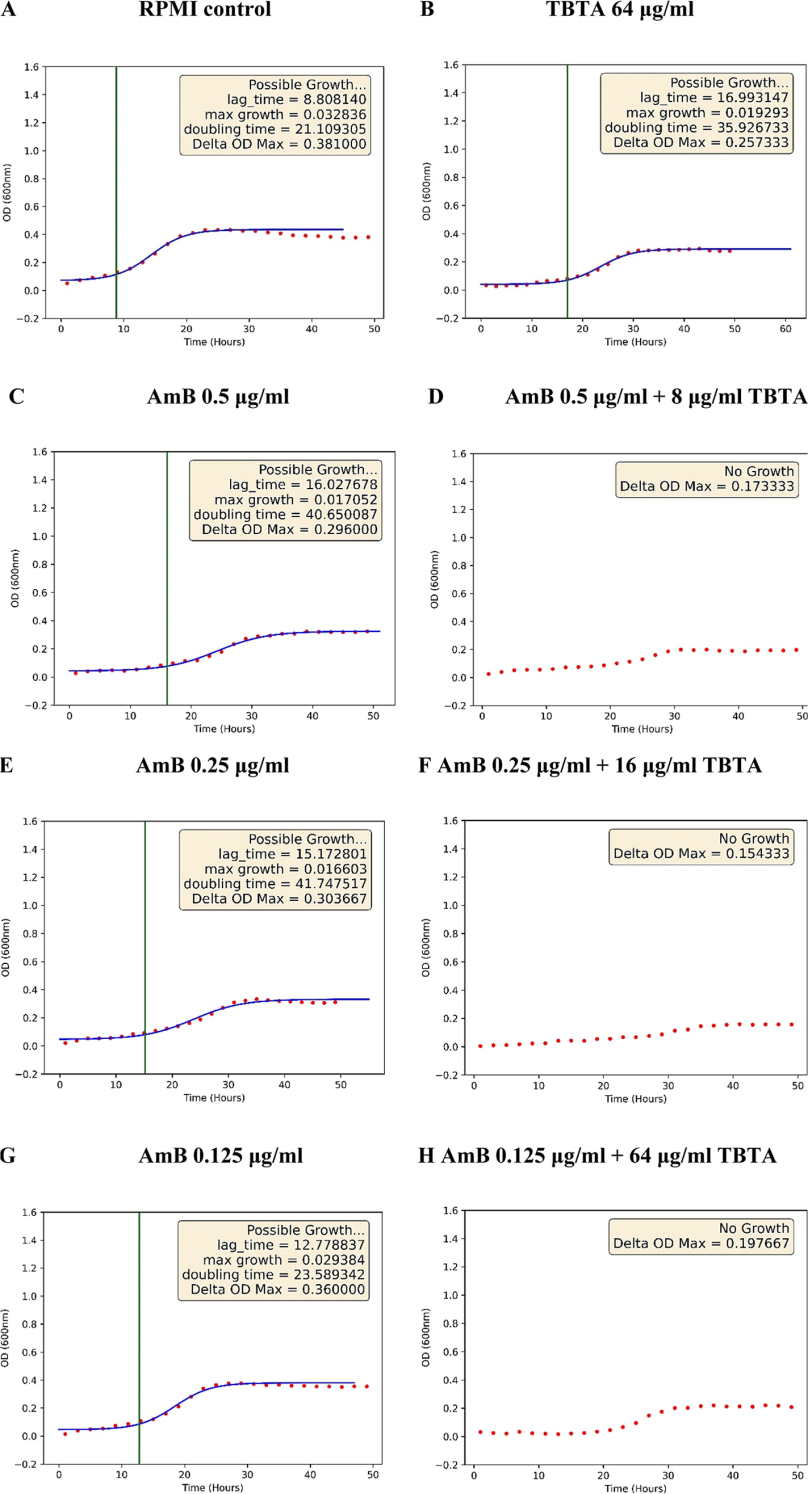
Growth curves for S. cerevisiae on RPMI media—Panel A, showing the impact of TBTA concentrated at 64 μg/mL—Panel B, AmB concentrated at 0.5 μg/mL—Panel C, 0.25 μg/mL—Panel E, 0.125 μg/mL—Panel G, as well as synergistic compositions thereof concentrated at AmB 0.5 μg/mL +8 μg/mL TBTA—Panel D, AmB 0.25 μg/mL +16 μg/mL TBTA—Panel F, and AmB 0.125 μg/mL +64 μg/mL TBTA—Panel H.

**Table 3:** ΣFIC Values for the AmB and TBTA Systems Yielding the Best Effects

probes	ΣFIC
AmB 0.5 μg/mL + TBTA 8 μg/mL	0.562
AmB 0.25 μg/mL + TBTA 16 μg/mL	0.375
AmB 0.125 μg/mL + TBTA 64 μg/mL	0.625

## Conclusions

The paper presents experiments utilizing spectroscopic methods and relevant calculations whose results clearly demonstrated that the synergistic interaction observed between the selected compound from the 1,3,4-thiadiazole group (TBTA) and AmB is related to the process of disaggregation of the AmB aggregates previously described in the literature. The measured CD, fluorescence anisotropy, and RLS spectra confirmed the impact of the 1,3,4-thiadiazole on the aggregation of AmB molecules. Fluorescence emission and fluorescence lifetime measurements performed for TBTA, AmB, and the synergistic composition aligned with the above results. Furthermore, measurements of DLS and RLS spectra taken in the same systems conclusively confirmed that size changes occur in the analyzed compositions.

The computational models corroborate the hypotheses posed on the basis of experimental results and provide a more in-depth insight into the interactions taking place in the synergistic system, wherein 1,3,4-thiadiazole molecules interact with AmB most readily in locations normally associated with the formation of the antibiotic’s aggregated structures, i.e., near its most hydrophobic regions. Another encouraging result is that the preferred binding sites of TBTA were found to resemble those of C1, which have also been shown to create synergistic compositions with AmB in solution. The additionally conducted biological study confirmed the exceptional antifungal potential of the presented composition, which far outperformed AmB alone. The addition of TBTA facilitated 4-fold reduction of the effective inhibitory concentration of the antibiotic in the most efficient synergistic system containing 0.25 μg/mL of AmB and 16 μg/mL of TBTA.

The paper presented above provides the first in-depth analysis of specific molecular interactions that contribute to our understanding of the clinical “golden standard” in the treatment of mycoses. In the future studies, we intend to continue biological investigations with a view to exposing other fungal organisms to the identified composition and exploring its potential in even greater detail. We also plan to focus on identifying other, potentially more effective molecules that might show similar synergistic effects and be capable of enhancing the therapeutic potential of this as well as other antibiotics. While the results available at this point are fragmentary, they do suggest that the interaction mechanism discussed above is general enough that it may very well be applicable also to other systems composed of two or more components.

## Supplementary Material



## Notes

Example fluorescence emission spectra for TBTA, AmB, and their synergistic composition in DMSO medium, analogous to the spectra in Figure 1, examples of fluorescence excitation spectra for TBTA, AmB, and their synergistic composition in DMSO medium (Panel A), and buffer PBS (Panel B), electronic absorption spectra for: TBTA, AmB, and the synergistic composition of TBTA + AmB, in DMSO—Panel A; Panel B—electronic absorption spectra for analogous systems in the PBS buffer medium (for absorbance below 0,1), concentration dependence of the absorbance ratio at 335 (aggregated form) and 408 nm (monomeric form) in the absorption spectrum at the synergistic composition of TBTA + AmB in PBS, and growth curves parameters of S. cerevisiae on RPMI media, with AmB and 2,4-dihydroxy-*N*-(5-methyl-1,3,4-thiadiazol-2-yl)­benzothioamide (TBTA) (PDF)
