# Supplementary material for: Advanced Spectroscopic and Theoretical Study and Assessment of Antimycotic Potential in a Synergistic Composition of a 1,3,4-Thiadiazole Derivative and Amphotericin B
Source: ACS Omega. 2025 May 22;10(21):21173–86. doi: 10.1021/acsomega.4c10113 (PMC12138605; doi:10.1021/acsomega.4c10113)
Supplement: Supplementary file 1 [file ao4c10113_si_001.pdf]

An Advanced Spectroscopic and Theoretical Study and Assessment of Antimycotic Potential in a Synergistic Composition of a 1,3,4-Thiadiazole Derivative and Amphotericin B

Lidia Ślusarczyk[1], Michaela Murzyniec[2], Mikołaj Gurba[2,3], Kamila Rachwał[4], Andrzej Górecki[5], James Hooper[2], Mariusz Gagoś[6], Arkadiusz Matwijczuk[1]**

1 Department of Biophysics, Faculty of Environmental Biology, University of Life Sciences in Lublin, Akademicka 13, 20-950 Lublin, Poland.

2 Faculty of Chemistry, Jagiellonian University, Gronostajowa 2, 30-387 Kraków, Poland.

3 Institute of Advanced Materials, Faculty of Chemistry, Wrocław University of Science and Technology, Wybrzeże Wyspiańskiego 27, 50-370 Wrocław, Poland.

4 Department of Biotechnology, Microbiology and Human Nutrition, Faculty of Food Science and Biotechnology, University of Life Sciences in Lublin, 8 Skromna Street, 20-704 Lublin, Poland.

5 Department of Physical Biochemistry, Faculty of Biochemistry, Biophysics and Biotechnology, Jagiellonian University, Kraków, 30-387, Poland.

6 Department of Cell Biology, Maria Curie-Sklodowska University, Akademicka 19, 20-033, Lublin, Poland.

Corresponding authors: lidia.slusarczyk@up.lublin.pl (L.Ś); arkadiusz.matwijczuk@up.lublin.pl (A.M.)

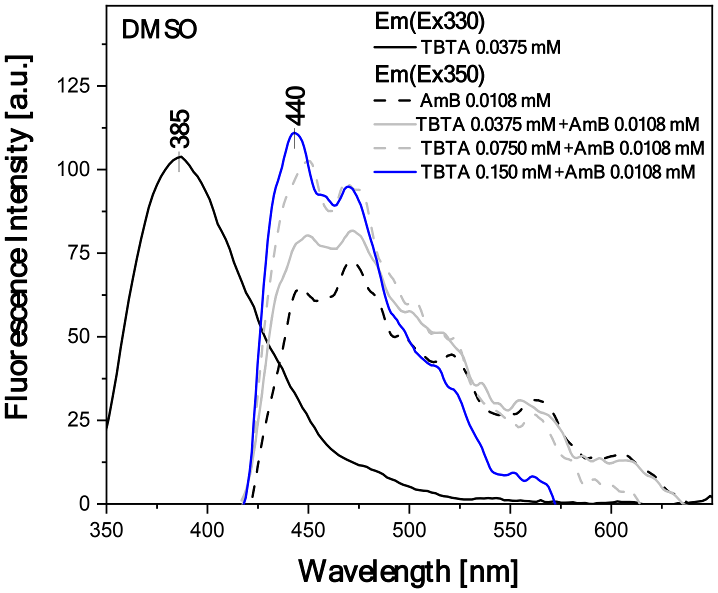

Figure SM1. Example fluorescence emission spectra for TBTA, AmB, and their synergistic composition in DMSO medium, analogous to the spectra in Figure 1.

A **B**

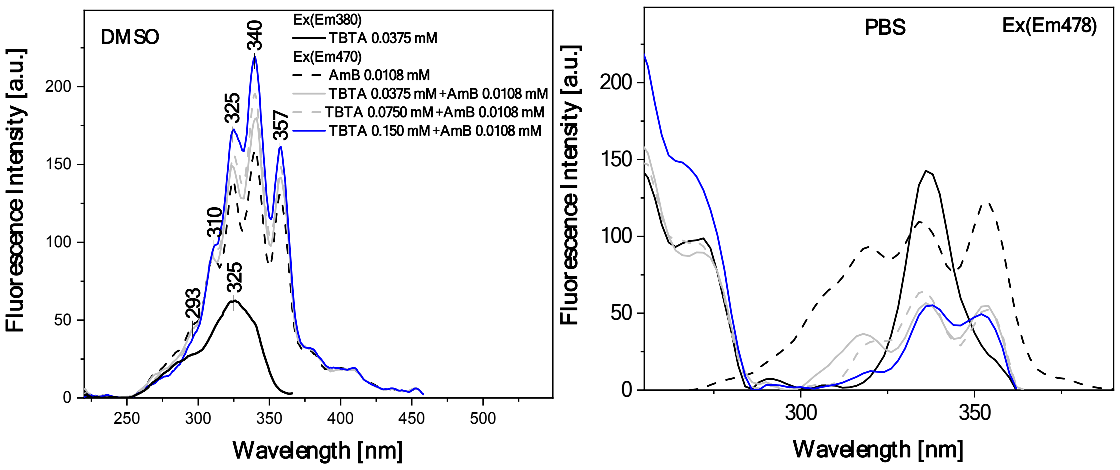

Figure SM2. Examples of fluorescence excitation spectra for TBTA, AmB, and their synergistic composition in DMSO medium (Panel A), and buffer PBS (Panel B).

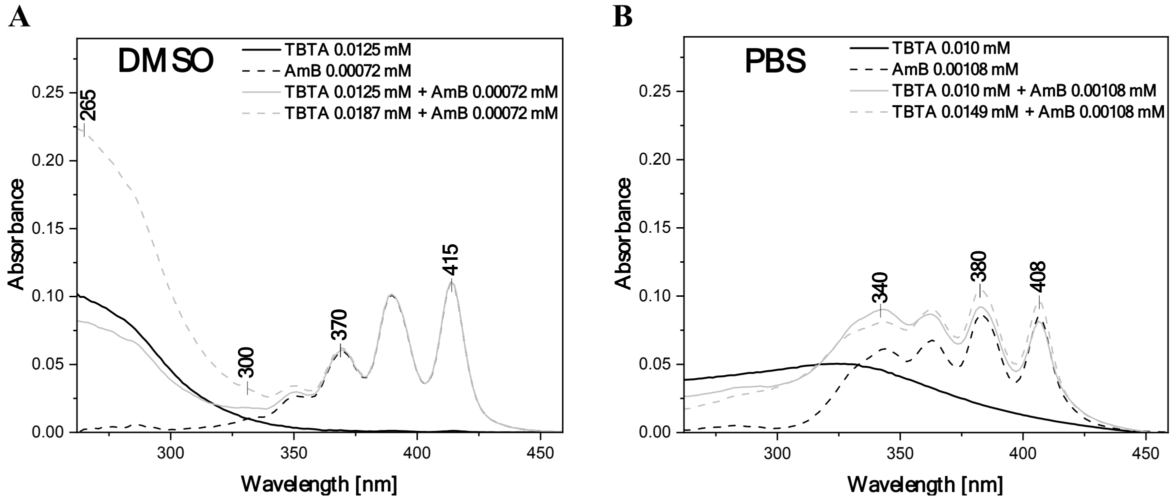

Figure SM3. Electronic absorption spectra for: TBTA, AmB, and the synergistic composition of TBTA + AmB, in DMSO - Panel A; Panel B – electronic absorption spectra for analogous systems in the PBS buffer medium (for absorbance below 0,1).

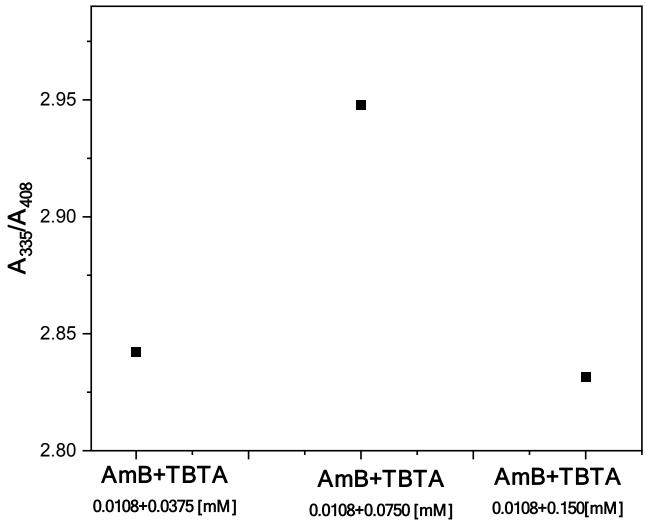

Figure SM4. The concentration dependence of the absorbance ratio at 335 (aggregated form) and 408 nm (monomeric form) in the absorption spectrum at the synergistic composition of TBTA + AmB in PBS.

Table SM1. Growth curves paramethers of *S. cerevisiae* on RPMI media, with Amphotericin B (AmB) and 2,4-dihydroxy-N-(5-methyl-1,3,4-thiadiazol-2-yl)benzothioamide (TBTA).

**Sample**		**Lag Time (hours)**	**Max Specific Growth Rate (1/hours)**	**Doubling Time (hours)**	**Max OD**	**Max OD (Median Filtered Data)**	**Min OD**	**Min OD (Median Filtered Data)**	**Delta OD (Median Filtered Data)**	**R^2**
**AmB µg/ml**	**TBTA µg/ml**									
RPMI control		8.81	0.033	21.11	0.432	0.432	0.051	0.051	0.381	0.997
	256				0.088	0.087	0.029	0.087	0	
	128				0.233	0.229	0.039	0.039	0.189	-
	64	16.99	0.019	35.93	0.292	0.290	0.027	0.032	0.257	0.995
	32	13.43	0.018	38.61	0.307	0.305	0.008	0.008	0.296	0.996
	16	9.59	0.027	25.21	0.322	0.321	0.013	0.013	0.308	0.997
	8	11.26	0.028	24.93	0.344	0.336	0.019	0.019	0.317	0.996
	4	7.51	0.029	23.49	0.338	0.335	0.032	0.032	0.303	0.995
	2	9.41	0.027	25.55	0.317	0.311	0.024	0.024	0.287	0.995
	1	9.56	0.029	23.82	0.323	0.309	0.024	0.024	0.285	0.995
4					0.068	0.062	0.038	0.061	0.001	
2					0.051	0.049	0.012	0.037	0.012	
**1**					**0.094**	**0.094**	**0.040**	**0.040**	**0.054**	
0.5		16.03	0.017	40.65	0.323	0.323	0.027	0.027	0.296	0.995
0.25		12.00	0.017	40.97	0.341	0.341	0.030	0.030	0.311	0.996
0.125		8.28	0.022	31.19	0.377	0.376	0.027	0.027	0.348	0.996
0.06		6.97	0.023	30.54	0.425	0.415	0.031	0.031	0.383	0.998
0.03		8.45	0.025	27.88	0.448	0.445	0.033	0.033	0.412	0.999
**0.5**	**8**				**0.199**	**0.198**	**0.025**	**0.025**	**0.173**	
0.25	8	12.71	0.009	77.49	0.267	0.257	0.008	0.009	0.247333	0.988
0.125	8	12.70	0.032	21.60	0.425	0.425	0.010	0.010	0.415	0.986
0.06	8	9.56	0.024	28.88	0.357	0.345	0.019	0.019	0.325	0.997
0.03	8	8.19	0.028	25.15	0.372	0.364	0.039	0.039	0.324	0.997
0.25	16				0.160	0.159	0.004	0.004	0.154	
0.125	16	4.90	0.015	47.22	0.383	0.355	0.010	0.0103	0.344	0.993
0.06	16	11.51	0.019	35.85	0.247	0.245	0.001	0.001	0.243	0.998
0.03	16	12.38	0.023	30.46	0.256	0.250	0.003	0.003	0.246	0.999
0.125	64				0.221	0.218	0.018	0.020	0.198	
0.125	128	31.42	0.021	32.35	0.275	0.273	0.027	0.027	0.246	0.981
